# Cardiovascular Access Gaps in High-Deprivation U.S. Counties With Premature CVD Mortality

**DOI:** 10.1016/j.jaccas.2026.109327

**Published:** 2026-09-02

**Authors:** Baryalay Khan, Hamid Raza, Furrukh S. Malik

**Affiliations:** a Department of Medicine, Vanderbilt University Medical Center, Nashville, Tennessee, USA; b Department of Statistics and Economics, Aalborg University, Aalborg, Denmark; c Division of Cardiovascular Medicine, Vanderbilt University Medical Center, Nashville, Tennessee, USA

**Keywords:** cardiac rehabilitation, cardiology workforce, cardiovascular mortality, deprivation, health equity, rural health

## Abstract

**Background:**

Premature cardiovascular disease (CVD) mortality in the United States is geographically uneven, but prevention planning requires identifying whether high-burden communities face limited cardiovascular care infrastructure.

**Methods:**

Beginning with an England-U.S. comparison, we identified a high-risk U.S. county tail, defined as counties in both the highest deprivation quintile and the top quartile of county-level mean premature CVD mortality. We characterized these counties by rurality, cardiometabolic risk factors, insurance, primary care capacity, cardiology workforce, and cardiac-service availability.

**Results:**

High-risk-tail counties were predominantly rural, had higher risk-factor burden and uninsurance, and had lower primary care and cardiovascular physician density. Most lacked local patient-care cardiovascular physicians, adult cardiology services, diagnostic catheterization, interventional catheterization, and cardiac rehabilitation.

**Conclusions:**

High-deprivation U.S. counties with excess premature CVD mortality represent a structural cardiovascular care gap. Geographically targeted prevention, workforce planning, and cardiac rehabilitation access should prioritize areas where premature mortality, deprivation, rurality, and limited cardiovascular infrastructure overlap.

## Graphical Abstract

**Figure:**
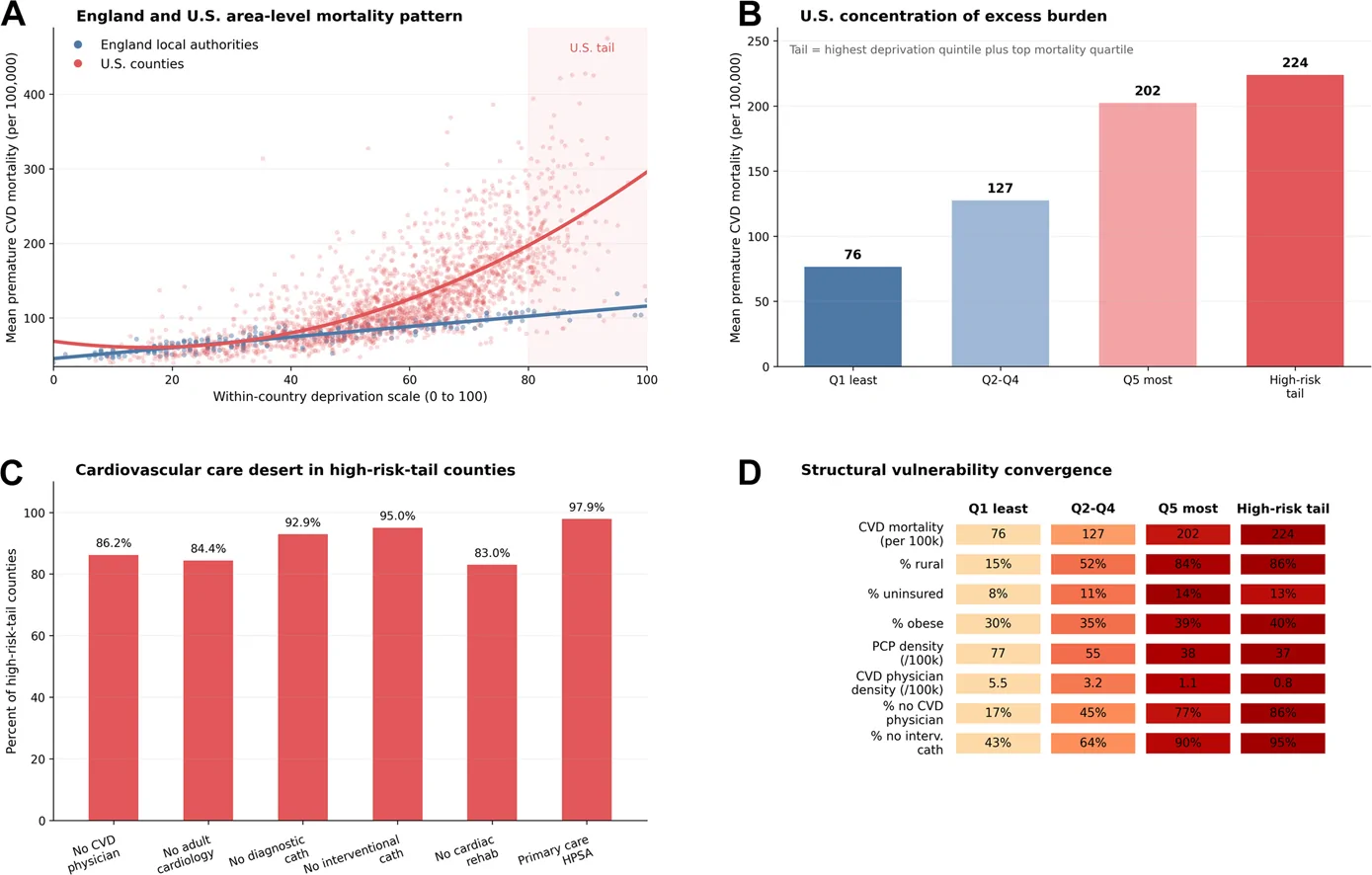

Cardiologists often encounter the downstream consequences of geographically uneven prevention and access, but county-level surveillance can identify where premature cardiovascular disease (CVD) mortality, deprivation, rurality, and limited cardiovascular infrastructure converge before patients reach advanced disease. Area deprivation is strongly associated with CVD outcomes,^1^ but the shape and concentration of that association are clinically important. A broadly distributed gradient suggests a population-wide prevention challenge, whereas a high-risk county tail—defined in this analysis as U.S. counties in both the highest deprivation quintile and the top quartile of county-level mean premature CVD mortality—identifies communities where mortality rises disproportionately and targeted resources may be most needed.

This Chapter Corner analysis asks a U.S.-focused surveillance question: are high-burden counties simply high-mortality areas, or do they also represent structurally vulnerable cardiovascular care environments? England was used as a contextual comparator because it has an established national deprivation measure, comparable mortality surveillance infrastructure, and sufficient geographic units for within-country gradient analysis. We did not assume that England's Index of Multiple Deprivation (IMD) and the U.S. Area Deprivation Index (ADI) are equivalent exposures. Instead, each measure was used to rank areas within its own country.

We then focused on the United States to characterize the counties in which premature CVD mortality, deprivation, rurality, cardiometabolic risk, and limited cardiovascular access converge.

## Methods

We assembled linked area-year data for lower-tier local authorities in England and counties in the United States from 2010 through 2020. The outcome was age-standardized premature CVD mortality before age 75 years.^2^^,^^3^ Deprivation was measured using IMD in England and ADI in the United States,^4^^,^^5^ averaged across available pre-2021 releases and rescaled from 0 to 100 within each country. Higher values represent greater relative deprivation within the same country only.

The analytic sample included 282 English local authorities and 2,049 U.S. counties with available mortality and deprivation data. U.S. counties without linkable mortality rates were generally smaller and more rural, consistent with suppression or instability of small-area mortality estimates.

For the U.S. structural profile, county-level data were linked to Rural-Urban Continuum Code rurality,^6^ smoking, obesity, diabetes, uninsurance, primary care physician density,^7^ primary care Health Professional Shortage Area (HPSA) designation,^8^ and Area Health Resources File (AHRF) 2019 to 2020 workforce and hospital-service variables.^9^ AHRF variables included 2018 patient-care CVD physician density and hospitals with adult cardiology services, diagnostic catheterization, interventional catheterization, cardiac surgery, electrophysiology, and cardiac rehabilitation. U.S. access analyses included all 2,049 counties with linkable AHRF variables; HPSA variables were linkable for 2,000 counties, including all 282 high-risk-tail counties. Missing access variables were not imputed.

We summarized county-level mean mortality for descriptive profiling. For annual area-year deprivation-mortality patterning, we used country-specific ordinary least-squares models with robust standard errors clustered by area and included quadratic terms to assess nonlinearity. The U.S. high-risk tail was defined as counties in the highest deprivation quintile and the top quartile of county-level mean premature CVD mortality. Analyses used publicly available, de-identified aggregate data and did not require institutional review board review.

## Results

**Figure 1:**
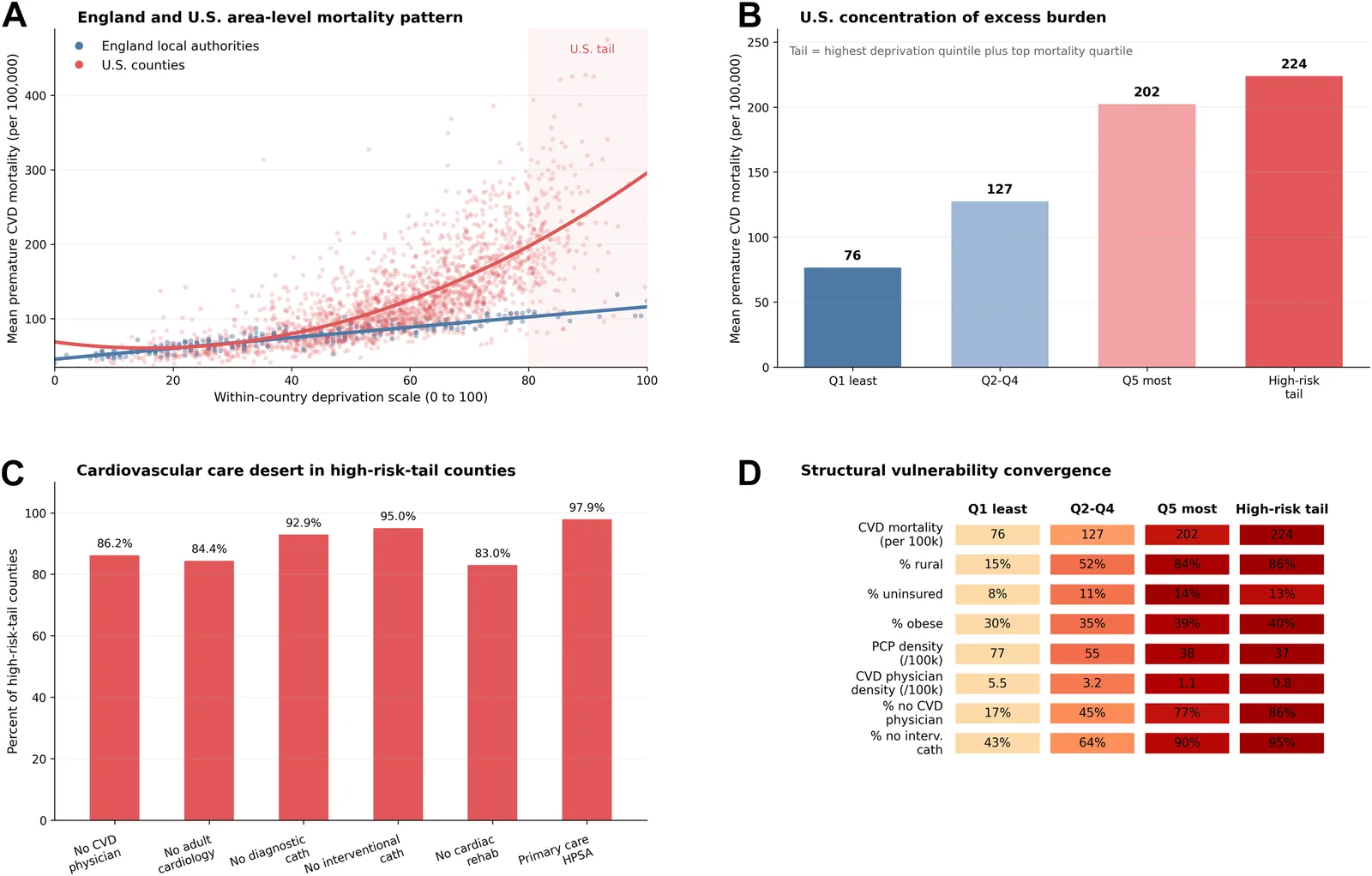
Cardiovascular Access Gaps in High-Deprivation U.S. Counties With Premature CVD Mortality (A) Actual area-level mean premature CVD mortality by within-country deprivation scale for England and the United States; fitted curves are country-specific quadratic fits of area-level mean mortality on within-country deprivation score. (B) County-level mean premature CVD mortality across U.S. deprivation groups and the high-risk tail. (C) The percentage of high-risk-tail counties lacking key cardiovascular or primary care resources. (D) Summary of structural vulnerability across U.S. county groups; darker shading indicates higher burden or lower workforce density. High-risk tail was defined as U.S. counties in the highest deprivation quintile and the top quartile of county-level mean premature CVD mortality. Data sources: CDC WONDER, Office for Health Improvement and Disparities Fingertips, USDA Rural-Urban Continuum Codes, County Health Rankings, HRSA Area Health Resources Files 2019 to 2020, and HRSA HPSA data. CVD = cardiovascular disease; HPSA = Health Professional Shortage Area; PCP = primary care physician.

Premature CVD mortality increased with deprivation in both England and the United States, but the U.S. distribution showed a much higher high-deprivation tail. At the area level, mean mortality in England increased from 54.7 deaths per 100,000 in the least deprived quintile to 96.5 deaths per 100,000 in the most deprived quintile. In the United States, mean county-level mortality increased from 76.5 deaths per 100,000 in the least deprived quintile to 202.2 deaths per 100,000 in the most deprived quintile. The high-risk tail contained 282 U.S. counties and had mean premature CVD mortality of 223.8 deaths per 100,000. These deprivation-mortality patterns and the definition of the high-risk tail are summarized in Figures 1A and 1B.

The rurality pattern differed sharply by country. In England, the most deprived local authorities were predominantly urban: 6.6% of the most deprived English authorities were rural, compared with 58.9% of the least deprived authorities. In the United States, the pattern was reversed. Among the most deprived U.S. counties, 83.5% were rural, compared with 14.8% of the least deprived counties. The high-risk-tail counties were 85.8% rural.

The most deprived U.S. counties had a markedly higher cardiometabolic and access burden than the least deprived counties. Smoking prevalence was 24.4% vs 16.0%, obesity 39.4% vs 30.1%, diabetes 16.6% vs 10.2%, and uninsurance 13.7% vs 8.1%. Primary care physician density was lower in the most deprived counties than the least deprived counties, 38.1 vs 77.3 per 100,000. Patient-care CVD physician density was also lower, 1.1 vs 5.5 per 100,000. A full profile of U.S. counties by deprivation group and high-risk-tail status is shown in Table 1.

**Table 1:** Profile of U.S. Counties by Deprivation and High-Risk-Tail Status

Measure	Q1 Least Deprived	Q2-Q4	Q5 Most Deprived	High-Risk Tail
Number of counties	454	1,225	370	282
Mean premature CVD mortality, per 100,000	76.5	127.5	202.2	223.8
Rural counties, %	14.8	52.4	83.5	85.8
Smoking, %	16.0	20.7	24.4	24.9
Obesity, %	30.1	35.3	39.4	39.8
Diabetes, %	10.2	12.9	16.6	16.8
Uninsured, %	8.1	11.0	13.7	13.1
Primary care physician density, per 100,000	77.3	55.3	38.1	36.5
Patient-care CVD physician density, per 100,000	5.5	3.2	1.1	0.8
No patient-care CVD physician, %	16.7	44.8	77.0	86.2
No interventional catheterization, %	43.2	63.6	90.3	95.0

High-risk tail was defined as U.S. counties in the highest deprivation quintile and the top quartile of county-level mean premature CVD mortality. Q5 most deprived includes all counties in the highest deprivation quintile; the high-risk tail is a subset of Q5. County-level mortality values are based on each county's mean mortality across available years.

CVD = cardiovascular disease.

Cardiovascular workforce and cardiac-service availability were particularly limited in the high-risk tail. Among high-risk-tail counties, 86.2% had no patient-care CVD physician, 84.4% had no hospital with adult cardiology services, 92.9% had no diagnostic catheterization availability, 95.0% had no interventional catheterization availability, 83.0% had no cardiac rehabilitation availability, and 97.9% had an active primary care HPSA designation.

## Discussion

This analysis identifies a concentrated U.S. cardiovascular equity problem. England provides context: deprivation was associated with higher premature CVD mortality, but the most deprived areas were largely urban and the mortality gradient was less concentrated. In the United States, high deprivation clustered with rurality, cardiometabolic risk burden, uninsurance, lower primary care density, and limited cardiovascular workforce and cardiac-service availability.

These findings are descriptive, not causal. Smoking, obesity, diabetes, uninsurance, and physician supply may be mediators or downstream correlates of structural deprivation rather than traditional confounders; adjusted models should therefore be interpreted descriptively. The main contribution is identifying where premature CVD mortality, deprivation, rurality, and access constraints converge.

This distinction is clinically important. A high-deprivation rural county with high premature CVD mortality, low primary care density, no local cardiovascular disease physician, and no cardiac rehabilitation availability presents a different prevention challenge than a deprived urban area with closer health care infrastructure. Cardiovascular equity surveillance should therefore move beyond national averages and average deprivation slopes toward identifying high-need localities where structural vulnerability is concentrated.

For cardiologists and ACC chapters, these findings support a geographically targeted cardiovascular prevention agenda. Potential responses include targeted risk-factor screening, blood pressure and lipid-control initiatives, telecardiology partnerships, cardiac rehabilitation referral and access expansion, and workforce planning that accounts for CVD burden rather than population size alone. State chapters may be particularly well positioned to identify high-burden counties and partner with health systems, public health agencies, and primary care networks.

Several limitations should be emphasized. First, this ecological analysis cannot support individual-level inference. Second, IMD and ADI are country-specific deprivation measures and were used only for within-country ranking, not direct equivalence. Third, AHRF workforce and cardiac-service variables were measured cross-sectionally from the 2019 to 2020 release and may not represent access conditions throughout the full 2010 to 2020 mortality period. Fourth, AHRF variables describe county-level service availability, not affordability, procedural volume, quality, travel time, or patient-level access.

## Conclusions

The U.S. excess burden of premature CVD mortality is concentrated in a high-deprivation county tail where rurality, cardiometabolic risk, uninsurance, and limited cardiovascular access converge. Cardiovascular equity surveillance should identify not only who is at risk, but where premature mortality and structural cardiovascular vulnerability overlap.

## Prior Presentation

Presented in part as a poster at the Tennessee Chapter of the American College of Cardiology Annual Meeting (November 14, 2025) and at the Global Health Summit at Belmont University (January 24, 2026), where it received awards at both venues.

### Data Availability

Source data were publicly available from the sources cited in the references. Derived analytic data and code can be made available by the corresponding author on reasonable request.

### Use of Artificial Intelligence

ChatGPT (OpenAI) was used for language editing, formatting, and figure refinement. The authors reviewed all content, verified all data values, and take responsibility for the final manuscript.

## Funding Support and Author Disclosures

The authors have reported that they have no relationships relevant to the contents of this paper to disclose.

**Take-Home Messages**

• The U.S. high-risk tail of premature CVD mortality is concentrated in high-deprivation counties that are predominantly rural and structurally underserved.
• Geographically targeted cardiovascular prevention and workforce planning should prioritize communities where premature CVD mortality, deprivation, rurality, and limited cardiac infrastructure overlap.
